# Identifying axial spondyloarthritis flares using the Evaluation of Ankylosing Spondylitis Quality of Life (EASiQoL) patient-reported outcome measure via an online portal: the FASTER study protocol

**DOI:** 10.1093/rap/rkaf107

**Published:** 2025-09-13

**Authors:** James A Prior, Helen Parsons, Kirstie L Haywood, Ian C Scott, Jake Grange, Alison Williams, Jon Packham

**Affiliations:** School of Medicine, Keele University, Staffordshire, UK; Rheumatology Department, Midlands Partnership University NHS Foundation Trust, Stafford, UK; Warwick Medical School, University of Warwick, Coventry, UK; Warwick Medical School, University of Warwick, Coventry, UK; School of Medicine, Keele University, Staffordshire, UK; Rheumatology Department, Midlands Partnership University NHS Foundation Trust, Stafford, UK; Rheumatology Department, Midlands Partnership University NHS Foundation Trust, Stafford, UK; Warwick Medical School, University of Warwick, Coventry, UK; Rheumatology Department, Midlands Partnership University NHS Foundation Trust, Stafford, UK; School of Medicine, Keele University, Staffordshire, UK; Rheumatology Department, Midlands Partnership University NHS Foundation Trust, Stafford, UK; Academic Unit of Population and Lifespan Sciences, University of Nottingham, Nottingham, UK

**Keywords:** axial spondyloarthritis, ePROM, EASiQoL, flare, patient-initiated follow-up

## Abstract

**Background:**

People with axial spondyloarthritis (axSpA) frequently experience increases in symptoms (flares) related to pain, stiffness and fatigue. Despite frequent use of the term ‘flare’ within axSpA, it is poorly defined and characterised. No widely accepted patient-reported outcome measure (PROM) exists that can identify axSpA flares or quantify their severity. However, the Evaluation of Ankylosing Spondylitis Quality of Life (EASiQoL) is a patient-derived axSpA-specific PROM with strong evidence of its ability to assess quality of life, an aspect affected by changes in symptoms.

**Objective:**

This study will look at the feasibility of identifying flares in people with axSpA through regular completion of an electronic (e) version of the EASiQoL.

**Methods:**

We will conduct a mixed-methods 12-month prospective cohort study. Up to 1000 people with axSpA from rheumatology departments and all members of the National Axial Spondyloarthritis Society will be invited to complete a baseline questionnaire containing several ePROMs, including the EASiQoL, via a bespoke online study portal. Of the estimated 1280 responders, all will receive e-mail reminders to complete monthly questionnaires for the following 10 months and a final 12-month follow-up questionnaire. Over the 12 months, a subsample of up to 40 participants who report a flare during this period will be invited to a qualitative interview. Their lived experience of ‘flare’ and how this was reflected in their EASiQoL scores will be explored.

**Potential impact:**

Collection of ePROM data sensitive to the presence of axSpA flares should provide readily accessible information to both patients and rheumatology teams to monitor disease course and develop more effective flare pathways in axSpA.

## Introduction

Axial spondyloarthritis (axSpA) causes chronic inflammation of the pelvis, hips and lower spine. One in every 200 people living with axSpA in the UK frequently experience increases in symptoms (flares) related to pain, stiffness and fatigue [1]. These flares are relatively common, with a minor or major flare experienced at a rate of 71.4 per 100 person-weeks [2]. Flares vary in duration, pattern and presentation, potentially driven by any number of different factors [3]. Reflecting this, patients may require different approaches and types of support to manage their flares, dependent upon the predominant symptom/causation, e.g. physiotherapy for increases in physical restriction, medical management for increases in inflammation or pain.

Traditionally, rheumatology departments request patients attend routine clinic follow-ups every 6 or 12 months, but to otherwise make contact when flares arise. However, this model of care can result in patients either attending unnecessarily, missing booked appointments when well or being unable to access services when unwell, increasing the financial and time burdens placed on both patients and rheumatology departments. To address this imbalance of service provision, the ‘Getting It Right First Time’ initiative has produced guidance on implementing patient-initiated follow-up (PIFU) in UK adult rheumatology services [4]. PIFU looks to reduce unnecessary routine appointments, with patients controlling the timing of clinical reviews as required. However, PIFU is a new process that is still being implemented across UK rheumatology departments, with many patients continuing to attend routine clinics. If the transition to PIFU is to maintain safe, high-quality services, then new pathways are required to ensure that patients are empowered to access services when needed, especially when experiencing a flare.

Since the COVID-19 pandemic, patients and clinicians have become accustomed to accessing services remotely, and the monitoring of health via electronic patient-reported outcome measures (ePROMs) is increasingly common and acceptable. The potential now exists for a new pathway to remotely monitor the health of patients with axSpA through the completion of ePROMs so that rheumatology departments can respond accordingly and specifically when meaningful changes occur. This will help ensure efficient, high-quality, safe care for the increasing number of outpatients entering PIFU. However, such monitoring is dependent on ePROMs being both relevant to the experience of flares and responsive to changes in health.

Despite the frequent use of the term ‘flare’ within axSpA terminology, there is currently no accepted definition or characterisation of what a flare is or any widely accepted PROMs that identify axSpA flare or quantify flare severity across the breadth of patient experience. The Evaluation of Ankylosing Spondylitis Quality of Life (EASiQoL) is a high-quality patient-derived axSpA-specific questionnaire that is reliably responsive to changes in quality of life across four health domains: physical function, disease activity, mood and social participation [5, 6], all aspects of health that patients report are impacted by flares. If the EASiQoL is indicative of flare presence/absence, then remote completion could be used to monitor patients and promptly initiate individualised care, potentially focusing on the most prominent area of disease impact. The aim of the Flares in Axial Spondyloarthritis—Treating with Effective Resources (FASTER) study is to establish the feasibility of identifying/quantifying disease flares in people with axSpA through the completion of an electronic, online version of the EASiQoL.

## Methods

This mixed-methods study will include a 12-month prospective cohort study collecting quantitative data from people with axSpA via an online portal (stage 1), with qualitative interviews being conducted over this period in a subset of the sample (stage 2).

### Stage 1: Integrating and testing the EASiQoL on a digital access platform

#### Design

This is a 12-month prospective longitudinal cohort study. Patients with axSpA will be invited to complete a baseline questionnaire containing several ePROMs housed in a bespoke online National Health Service (NHS)-based study platform (the ‘FASTER Portal’). Baseline data collection will be followed by e-mail invites to complete brief monthly questionnaires for 10 months, followed by a longer 12-month end-of-study questionnaire. A ‘flare’ questionnaire will be available for participants to complete at any time. Ethical approval for stages 1 and 2 was granted by the Harrow Research Ethics Committee (24/PR/1172).

#### Sample and recruitment

People with axSpA will be recruited from February 2025 to July 2025. Approximately 1000 patients will be invited from each of 16 NHS Trust rheumatology departments across England and Wales (axSpA being defined clinically) and all members of the UK-wide National Axial Spondyloarthritis Society (NASS) (self-reported axSpA) will also be invited. Rheumatology departments will have the flexibility to approach their patients in-clinic or via post or e-mail invites. NASS members will receive an e-mail advertising the study and inviting them to take part.

Invitees will be asked to follow a weblink or use a QR code that is in their invitation letter. This will take them to the FASTER Portal. Upon entry, an electronic version of the patient information sheet (PIS) and consent form will be visible. Upon consent the patient will create a unique login, verify their portal account via their e-mail and then complete the baseline questionnaire. If potential participants fail to initially respond to their study invite, reminder letters/e-mails will be sent 1 and 2 weeks later.

#### Surveys and outcome measures

Baseline data collection will include demographic and clinical information such as year of birth, gender, disease duration and current treatment(s) (i.e. biologics). Several generic, domain and disease-specific ePROMs will also be completed (Table 1); specific ePROM completion will be dependent on the data collection time point. Additionally, if participants are experiencing a disease flare, this will be assessed by a yes/no question relating to the presence of a flare, participant-defined severity as ‘mild’, ‘moderate’ or ‘severe’ and the Aberdeen axSpA flare questionnaire.

**Table 1.** FASTER study data collection tools and time points.

Domain	Assessed by	Questionnaire time points					
		Baseline	Months 2–5	Month 6	Months 7–11	Month 12	Flare
Participant characteristics							
Demographics	Self-report	X					
Height	Self-report	X					
Weight	Self-report	X				X	
General health							
Health transition	Global impression of change [13]		X	X	X	X	X
Pain	Numerical rating scale (NRS)	X	X	X	X	X	X
Mental well-being	Warwick-Edinburgh Mental Wellbeing Scale (WEMWBS) [14]	X		X		X	X
Societal impact	Social Functioning Questionnaire (SFQ) [15]	X		X		X	X
Comorbidities	Self-reported inflammatory conditions	X				X	
	ACR criteria for fibromyalgia [16]	X				X	
Self-efficacy	6-item Self-Efficacy for Managing Chronic Disease scale [17]	X				X	
Disease specific							
Treatment	Self-report drug treatments	X				X	
Disease duration	Self-report (determined from year of diagnosis)	X					
Time to diagnosis	Self-report (period of symptoms before diagnosis)						
Quality of life	Evaluation of Ankylosing Spondylitis Quality of Life (EASi-QoL) [5, 6]	X	X	X	X	X	X
Work	Ankylosing Spondylitis Work Instability Scale (AS-WIS) [18]	X				X	
axSpA flare	Self-report (presence/severity)	X	X	X	X	X	X
	Aberdeen axSpA flare questionnaire	X				X	X
Fatigue	Warwick Axial Spondyloarthritis Fatigue and Energy questionnaire (WASTEd) [19]	X		X		X	X
Disease activity	Bath Ankylosing Spondylitis Disease Activity Index (BASDAI) [20]	X	X	X	X	X	X
Function	Bath Ankylosing Spondylitis Functional Index (BASFI) [21]	X		X		X	X
Global health	Bath Ankylosing Spondylitis Patient Global Score (BAS-G) [22]	X				X	

#### Data collection portal

Data will be collected and securely stored using the Haywood Arthritis Portal (HAP), a secure online co-designed system that collects ePROM data to inform the routine care of patients with inflammatory arthritis at the Midlands Partnership University NHS Foundation Trust [7]. A study-specific Portal (‘FASTER Portal’) located within the HAP was developed to collect research data. This hosts the PIS, allows collection of informed consent and creates reminder e-mails to prompt the participant to enter routine (monthly) data collection, with further reminders 1 and 2 weeks later if the survey remains uncompleted. While participants can view their ePROM scores on the portal (accompanied by guidance on score interpretation) and may choose to share these with their clinical team, clinical teams will not have direct access to study data. All research data collected by the portal will be available for download to the study team for anonymised data analysis.

#### Sample size

As the study aim is to evaluate the feasibility of identifying disease flares in patients with axSpA completing an electronic version of the EASiQoL, a formal power calculation was not possible. To achieve the study aims, a sample of people with axSpA who have a flare during follow-up is required. To do this, we intend to invite at least 1000 patients with axSpA from 16 NHS rheumatology department clinical sites across England and Wales and all members on NASS’s e-mail distribution list (≈6000+). Clinical sites are free to contact all patients who meet the inclusion criteria to reach individual targets. From the NASS we expect ≈1000 to express an initial interest, meaning we will approach 2000 people. Based on previous response rates [8], we estimate 1280 participants (64%) will complete the baseline questionnaire. Assuming loss to follow-up of 23% [6] over 12 months, our final sample will be 986. Of our retained sample, we estimate 36% (*n* = 355) will experience either a minor or major flare over the 12-month period [2]. We estimate that this will give us sufficient data on a wide range of patients with axSpA to allow for exploratory models to be constructed. A total of 250 participants will be required to support the comparative evaluation of measures [9].

#### Statistical analysis

Descriptive statistics of baseline variables will be constructed, along with summaries of outcome scores at each time point, along with any missingness. To establish if each EASiQoL domain scores or the Aberdeen axSpA flare questionnaire can be used to identify the presence of a flare, we will assess the following via descriptive statistics (where data allows): if self-reported flare severity is associated with a particular outcome score, if self-reported flare severity is associated with a change in scores from previous or subsequent non-flare score and, for the EASiQoL, if self-reported flare severity is associated with each individual domain score.

The absolute measurement error of the EASiQoL and Aberdeen axSpA flare questionnaire will be estimated by calculating the standard error of measurement (s.e.m.) and smallest detectable change (SDC) for participants who self-report small changes in their health. The minimal important change (MIC) will be estimated as the mean change in those reporting any flare, as well as those experiencing a minor flare, if numbers allow. A receiver operating characteristics (ROC) curve will be used to establish the most accurate cut point in scores for each outcome. We will consider an area under the ROC curve >0.7 as evidence that the outcome is sufficiently discriminatory and able to identify flares [10]. We will also examine the cohort for patterns of flaring and/or disease trajectories. This may include, for example, describing flare incidence and patterns as well as the identification of variables needed to differentiate between participants with self-reported well- controlled, mild, moderate or severe flares and uncontrolled disease, potentially using methods such as generalised linear mixed models. Additionally collected PROMs will allow us to further examine construct validity (both convergent and divergent) against each of the four EASiQoL domains and the Aberdeen axSpA flare questionnaire.

### Stage 2: Are changes in EASiQoL domain scores reflective of the self-reported flare experience?

#### Design

A sample of participants from stage 1 who report experiencing a flare will be invited to participate in a semi-structured online interview (the interview topic guide is presented in Supplementary Data S1). The aim of this will be to better understand participants lived experience of axSpA-related flares, the perceived driver of their flare and to explore how well scores on the EASiQoL reflect their ‘flare’ experience (i.e. a change in mental health domain score is corroborated by the patient as a change in mood).

#### Sample and recruitment

Participants will be invited if they have self-reported a flare in a recent stage 1 questionnaire (ideally within 2 weeks) and consented to be contacted about follow-up questioning as part of the study consent process. We estimate requiring up to 40 participants, to ensure sufficient range of data across the different flare presentations (mild, moderate, severe) and different EASiQoL domains [11]. We will purposively sample participants to ensure a range of characteristics related to gender, age and ethnicity. To ensure interviews are not influenced by knowledge of EASiQoL scores, participants will be blinded to these scores at the start of the interview; these will be revealed during the interview to allow more focused/reflective questioning around their health experience.

#### Data collection

All participants will provide informed consent prior to the interview via a signed consent form or, where this is not possible, an e-mail explicitly stating their ‘consent to take part’. Prior to commencing an interview, consent will also be verbally confirmed. Interviews will be conducted via telephone or video conferencing software (Microsoft Teams/Zoom). We anticipate that interviews will last 1–2 h and content will be digitally audio recorded (subject to participant permission). A semi-structured approach to the interviews will support participants in exploring their experience of axSpA and flare, what they consider to be a ‘flare’ and their experience of this and how well their EASiQoL scores reflect this experience.

#### Choice of analysis

Data collected from the interviews will be pseudonymised and transcribed verbatim. A reflective thematic analysis will be undertaken [12], informing the development of codes and then key themes that accurately reflect the data, align with the research objective and provide an understanding of the participants lived experiences. Interpretation will include similarities and differences within transcripts and across the whole dataset. NVivo 12 (Lumivero, Denver, CO, USA) will be used to help manage the data.

## Discussion

The principal output from the FASTER study will be to provide data related to the ability of the EASiQoL to identify the presence and severity of axSpA flares using regularly collected quantitative data through an electronic platform. As such, this study explores the potential of a new way of triaging rheumatology services in the NHS, reducing clinical pressures and supporting transition to models of individualised patient-centred care such as PIFU. Collection of ePROMs data, which changes in response to the presence of axSpA flares, would provide readily accessible information to both the patient and rheumatology multidisciplinary team to monitor disease course and initiate tailored advice or care pathways more promptly by reducing uncertainty in the role of axSpA. Such high-quality, psychometrically sound information is vital as increasing numbers of outpatients transition to patient-initiated care, requesting contact with rheumatology services at times of need. By empowering patients to self-monitor their disease, patients could be remotely triaged to attend the appropriate aspects of outpatient services when required, or even just arrange for further clinical tests. Such a process may improve efficiency and reduce unnecessary routine reviews within rheumatology services. This work would also bring the added benefit of new, longitudinal outcome measures data available in accessible, easily understandable, non-specialist outputs for both the patient and clinical team. Although this is being tested without concurrent clinical assessment data, potentially limiting the scope of any such triage process, it should be able to identify patient characteristics that might influence flare reporting and patient groups where patient-initiated review may be less successful. Positive results from the FASTER study will lay the foundation to test use of the EASiQoL as a method to prompt people with worsening axSpA symptoms to contact their rheumatology department where a triage process will guide them to the most appropriate care.

## Supplementary Material

rkaf107_Supplementary_Data
