# Supplementary material for: Identifying axial spondyloarthritis flares using the Evaluation of Ankylosing Spondylitis Quality of Life (EASiQoL) patient-reported outcome measure via an online portal: the FASTER study protocol
Source: Rheumatol Adv Pract. 2025 Sep 13;9(4):rkaf107. doi: 10.1093/rap/rkaf107 (PMC12512124; doi:10.1093/rap/rkaf107)
Supplement: rkaf107_Supplementary_Data [file rkaf107_supplementary_data.docx]

**Supplementary Data S1. FASTER study stage 2 – Interview topic Guide**

***Approx time of interview: 1-2hrs***

***Interview Objective***

To understand participants’ lived experience of their axial spondyloarthritis (axSpA)-related flare, the perceived driver of their flare, and how well scores across the individual domains of the EASi-QoL reflect their flare experience (axSpA).

**Sample**

Approximately 40 participants will be interviewed via telephone call or through use of video conferencing software. The participants will be recruited from those who have already been recruited in stage 1 of the FASTER study and who consented to further contact.

**Selection criteria**

- English speaking
- FASTER study stage 1 participant who consented to further contact
- Participant indicated in their most recent FASTER study questionnaire that they were experiencing a recent flare of their axSpA, having previously been not flaring
- Cognitively capable of participating in and emotionally coping with the interview

**Introductions, consent**

- Introductions
- Inform participant that the session is to be recorded and remind them that their testimonial will be anonymised during the transcription process.
- Confirmation that the interviewee has read and become acquainted with the participant information sheet (PIS) and consents to take part in the study.
- Inform patient that the interview could take between 1-2 hrs, that if a break is needed, they should just ask and that the interview will be roughly structured as follows:
  - Background information about axSpA and the FASTER study
  - Exploration of participants understanding of what an axSpA ‘flare’ is
  - Participant’s experience during their last flare
  - After informing the participant of their EASi-QoL score, how does this match (or not) to the participant’s experience during their last flare
  - Exploration of participant’s opinions around online data collection to monitor people’s axSpA flares

**Background Information, introducing research**

- Description of axSpA: Axial spondyloarthritis, also known as ankylosing spondylitis
  - - For the most part I’ll call it axial spondyloarthritis and axSpA
  - an inflammatory arthritis found in around 1 in 200 people in the UK
  - Primarily affects the lower back, causing chronic back pain and changes in the spine which can lead to reduced range of ability.
  - People with axSpA commonly experience flares, which can be defined as *“a period of time when your axSpA symptoms feel worse”*
  - Clearly identifying when someone is flaring remains difficult and patients are often unsure when they should consult healthcare because of it
- **This research is part of a study looking to better understand how a flare affects a person with axSpA, their perceived drivers of the flare, and to explore how well scores on the EASi-QoL reflect their ‘flare’ experience.**
- The first part of the study is collecting data through online questionnaires (as you’ve already been doing)
- This interview part of the study is to understand in greater detail whether the data collected from the questionnaire when you were flaring corresponds to how you actually felt the flare was impacting you
- We want to know whether the difficult health aspects measured by the EASi-QoL reflected your health at the time
- Do you have any questions at this stage?

**Flare understanding**

- ***What is living with axSpA like for you?***
- ***What happens if your axSpA gets worse?***
- ***You have recently experienced a ‘flare’ of your axSpA - how would you describe a flare of axSpA to someone else?***
- ***What do you think caused your most recent flare?***
  - Do you think that different things ‘trigger’ your flare?
  - Were there any other reasons why your health may have been worse during the period you thought you were having a flare?
- On average how often do you experience a flare of you axSpA?
- Do you find you are affected in similar or different ways each time?

**Flare experience**

- ***How severe would you say your last flare was (at the time of completing your last FASTER study questionnaire)?***
  - Are you still experiencing that flare?
- ***What effect did the flare have on your daily life? (Prompts: work, relationships, family)***
- ***Did you/ do you make any changes to your daily routine when you are experiencing a flare?***
- Was there a particular aspect of your health that was more affected by your flare?
  - Can you remember what symptoms you were experiencing?
  - Did you experience problems in more than one distinct way?
  - How easy is it for you to distinguish which aspects of your health are affected?
- ***How did you/ do you deal with/ manage your flare?***
- ***How do you look after yourself when you are experiencing a flare?***
- ***What works best for you to manage a flare?***
- ***Did you consult a healthcare professional about your last flare?***
  - What motivated you (or not) to seek medical attention?
  - If you saw a healthcare professional, can you recall what happened?
  - What do you think would be (have been) helpful to help you manage your current flare?
  - Is there a particular member of the rheumatology team who you feel might be most useful to see at this time? (Dr, physio, nurse, psychologist etc.)
  - Are flares discussed during your ‘usual’ visits with healthcare professionals? (e.g., physiotherapists, doctors?)
  - ***What are the things that you would like a healthcare professional to ask you about when discussing flares?***

**Participant made aware of their EASi-Qol score (and change in scores since previous non-flare score status)**

- In light of our previous discussions, how well do you feel your scores reflect how you felt during your flare?

**Online data collection**

- How would you feel if your Rheumatologist asked you to regularly complete a questionnaire so your flare activity could be monitored?
- How often would you like to provide such information online?
- How could your flare be better identified and managed?

**Interview wrap-up**

- That brings us to the end of the interview! Thank you so much for your time, it’s very much appreciated.
- Do you have any final questions?
- Thank you very much for your time and please do keep completing the FASTER questionnaires, your continued input is very much appreciated
